# Refinement of prognosis and the effect of azacitidine in intermediate-risk myelodysplastic syndromes

**DOI:** 10.1038/s41408-021-00424-4

**Published:** 2021-02-11

**Authors:** Konstantinos Liapis, Vasileios Papadopoulos, George Vrachiolias, Athanasios G. Galanopoulos, Menelaos Papoutselis, Sotirios G. Papageorgiou, Panagiotis T. Diamantopoulos, Vassiliki Pappa, Nora-Athina Viniou, Alexandra Kourakli, Dimitris Τsokanas, Theodoros P. Vassilakopoulos, Eleftheria Hatzimichael, Eleni Bouronikou, Maria Ximeri, Charalambos Pontikoglou, Aekaterini Megalakaki, Panagiotis Zikos, Panayiotis Panayiotidis, Maria Dimou, Stamatis Karakatsanis, Maria Papaioannou, Anna Vardi, Flora Kontopidou, Nikolaos Harchalakis, Ioannis Adamopoulos, Argiris Symeonidis, Ioannis Kotsianidis

**Affiliations:** 1 Department of Hematology, Democritus University of Thrace Medical School, Alexandroupolis, Greece; 2 Department of Clinical Hematology, Georgios Gennimatas Hospital, Athens, Greece; 3 Second Department of Internal Medicine, Attikon University General Hospital, Athens, Greece; 4 First Department of Internal Medicine, National and Kapodistrian University of Athens, Athens, Greece; 5 Greece Department of Internal Medicine, University Hospital of Patras, Rio, Greece; 6 Department of Hematology, Laikon General Hospital, National and Kapodistrian University of Athens, Athens, Greece; 7 Department of Hematology, University Hospital of Ioannina, Ioannina, Greece; 8 Department of Hematology, University Hospital of Larissa, Larissa, Greece; 9 Department of Hematology, University General Hospital of Heraklion, Voutes, Heraklion, Greece; 10 Department of Hematology, Metaxa Oncology Hospital, Piraeus, Greece; 11 Department of Hematology, Aghios Andreas General Hospital, Patras, Greece; 12 First Propedeutic Department of Internal Medicine, National and Kapodistrian University of Athens, Athens, Greece; 13 Department of Hematology, Sotiria General Hospital, Athens, Greece; 14 Department of Hematology, AHEPA University Hospital, Thessaloniki, Greece; 15 Department of Hematology and Stem cell Transplantation, Georgios Papanicolaou General Hospital, Thessaloniki, Greece; 16 Second Department of Internal Medicine, National and Kapodistrian University of Athens, Hippokratio General Hospital, Athens, Greece; 17 Department of Hematology and Bone Marrow Transplantation Unit, Evangelismos Hospital, Athens, Greece; 18 Department of Hematology and Thalassemia, Kalamata General Hospital, Kalamata, Greece

**Keywords:** Translational research, Myelodysplastic syndrome

Dear Editor,

The introduction of the revised International Prognostic Scoring System (IPSS-R) improved our ability to predict outcomes in patients with myelodysplastic syndromes (MDS)^1^. A major limitation of the IPSS-R is the large heterogeneity within the intermediate-risk category (IPSS-R 3.5–4.5). Recent evidence indicates variable outcomes of intermediate-risk patients and the need for additional risk factors to refine prognosis and guide therapeutic interventions^2^.

There are crucial questions about intermediate-risk MDS that need to be answered. Particularly important is whether patients of this category should be considered as having lower-risk or higher-risk disease. Although it was initially suggested that these patients should be placed in the lower-risk group regarding their potential therapeutic management^1^, subsequent analysis argued that the cutoff point between higher-risk and lower-risk MDS should be set at 3.5^3^. This immediately poses the question of whether azacitidine (AZA) should be used in intermediate-risk IPSS-R patients since AZA has been licensed by most health authorities for higher-risk MDS^4^.

Uncertainty or ambiguity about the role of AZA can lead to inaction by clinicians. Identifying groups of patients likely to benefit from AZA can help address this crucial question. There have been few systematic studies in IPSS-R intermediate patients to identify statistically significant clinical factors that predict survival and facilitate decisions about therapy. We aimed to identify risk factors that put patients at high risk for death and transformation to AML and explore the real-life AZA effectiveness in intermediate-risk MDS.

We drew our study population from the Hellenic National Registry of Myelodysplastic and Hypoplastic Syndromes which includes 2972 patients diagnosed with MDS, chronic myelomonocytic leukemia, and low blast-count acute myeloid leukemia (AML) between 1986 and 2016. A total of 468 patients (326 men; 142 women) aged 40.0–92.0 years (median, 73.0) with intermediate-risk IPSS-R were identified. The characteristics of the patients are summarized in Supplementary Table S1.

The primary outcomes were overall survival (OS) and leukemia-free survival (LFS). We analyzed the effects of the following factors: age; sex; hemoglobin; white-cell count; platelets; serum ferritin; lactate dehydrogenase; β2-microglobulin; estimated glomerular filtration rate (eGFR); WHO classification; cytogenetics; peripheral-blood and bone-marrow blasts; dyspoiesis; marrow cellularity; myelofibrosis; and transfusion dependency. We also asked if the Endothelial Activation and Stress Index (EASIX), a recently introduced biomarker, might be predictive of survival in intermediate-risk MDS. As in previous reports, we used the log2-transformed index^5,6^. We calculated survival according to the Kaplan–Meier method, and used a Cox proportional-hazards model as well as a decision-tree classification model to perform an adjusted analysis of survival (see Supplementary Information for a full description of statistical analysis methods).

Median follow-up was 51.0 months (range, 41.6–60.4), during which 220 (47.0%) patients died. AML developed in 150 patients (38.6%). Median OS was 31.0 months (95% confidence interval [CI] 26.6–35.4) and median LFS 26.0 months (21.5–30.5) (Supplementary Table S2). According to univariate analysis, age >70 years, male sex, performance status ≥2, transfusion dependency, eGFR <45 mL/min/1.73 m^2^, β2-microglobulin >3.0 mg/L, log2 EASIX, circulating blasts, and excess marrow blasts were associated with inferior OS (Table 1; Supplementary Fig. S1). On multivariate analysis, circulating blasts ≥1% (*p* = 0.003), age >70 years (*p* = 0.001), IPSS-R > 3.5 (*p* = 0.040), and log2 EASIX > 0.179 (*p* = 0.036) emerged as significant independent prognostic factors for OS (Table 1). Significant univariate risk factors associated with LFS included age, sex, performance status, transfusion dependency, circulating and bone-marrow blasts, eGFR, and β2-microglobulin (Table 1; Supplementary Fig. S2) but, in the multivariate analysis, only circulating blasts (hazard ratio [HR] 1.51, 95% CI 1.10–2.08; *p* = 0.011) and age >70 years (HR 1.66, 95% CI 1.25–2.21; *p* < 0.001) remained significant (Table 1).

**Table 1:** Results of univariate and multivariate analysis of clinical and laboratory predictors of outcome in the entire cohort of patients with intermediate-risk IPSS-R.

Variable	No. of patients with variable (%)	Overall survival				Leukemia-free survival			
		Univariate analysis		Multivariate analysis		Univariate analysis		Multivariate analysis	
		*P* value	Hazard ratio (95% CI)	*P* value	Hazard ratio (95% CI)	*P* value	Hazard ratio (95% CI)	*P* value	Hazard ratio (95% CI)
Age >70 years	293 (62.7)	<0.001	1.77 (1.37–2.29)	0.001	1.65 (1.24–2.20)	<0.001	1.64 (1.27–2.12)	<0.001	1.66 (1.25–2.21)
Female sex	142 (30.3)	0.013	0.71 (0.54–0.93)	NS		0.026	0.74 (0.56–0.96)	NS	
Cardiac comorbidity^a^	93 (21.7)	0.278	1.09 (0.94–1.26)	NS		0.274	1.09 (0.94–1.27)	NS	
Pulmonary comorbidity^a^	47 (11.0)	0.360	1.20 (0.81–1.77)	NS		0.481	1.16 (0.77–1.73)	NS	
Renal comorbidity^a^	17 (4.0)	0.251	1.43 (0.78–2.61)	NS		0.430	1.28 (0.70–2.34)	NS	
MDS-CI intermediate or high	145 (33.9)	0.072	1.27 (0.98–1.63)	NS		0.115	1.23 (0.95–1.60)	NS	
WHO performance status >1	55 (14.2)	<0.001	1.73 (1.33–2.25)	NS		<0.001	1.65 (1.26–2.16)	NS	
IPSS-R score >3.5 (versus 3.5)	315 (67.3)	0.039	1.37 (1.04–1.80)	0.040	1.37 (1.02–1.84)	0.057	1.35 (1.04–1.75)	NS	
IPSS intermediate 2 or high risk	78 (16.7)	0.295	1.19 (0.86–1.66)	NS		0.311	1.18 (0.85–1.64)	NS	
Log2 EASIX score^b^	Assessed as continuous variable	0.001	1.27 (1.10–1.46)	ND		0.006	1.22 (1.06–1.41)	ND	
Log2 EASIX score >0.179^b^	303 (71.1)	0.009	1.46 (1.10–1.94)	0.036	1.39 (1.02–1.89)	0.059	1.32 (0.99–1.76)	NS	
Cytogenetics intermediate or poor according to IPSS-R	119 (26.3)	0.059	1.32 (0.99–1.75)	NS		0.085	1.28 (0.97–1.69)	NS	
FPSS score ≥ 2	183 (72.9)	0.356	1.18 (0.82–1.69)	NS		0.543	1.12 (0.78–1.62)	NS	
Transfusion dependency^c^	178 (51.3)	0.009	1.47 (1.11–1.96)	NS		0.024	1.39 (1.04–1.85)	NS	
Hemoglobin level	Assessed as continuous variable	0.209	0.96 (0.90–1.02)	NS		0.448	0.98 (0.92–1.04)	NS	
Hemoglobin <10 g/dL	284 (60.8)	0.376	0.89 (0.70–1.15)	NS		0.655	0.94 (0.73–1.21)	NS	
Absolute neutrophil count	Assessed as continuous variable	0.052	1.03 (1.00–1.07)	NS		0.243	1.02 (0.99–1.06)	NS	
Absolute neutrophil count <1.5 × 10^9^/L	214 (45.9)	0.568	0.93 (0.73–1.19)	NS		0.926	1.01 (0.79–1.29)	NS	
Platelet count	Assessed as continuous variable	0.571	1.00 (0.99-1.01)	NS		0.619	1.00 (0.99–1.01)	NS	
Platelet count <100 × 10^9^/L	223 (47.6)	0.545	1.08 (0.85–1.37)	NS		0.805	1.03 (0.81–1.32)	NS	
Bone-marrow blast percentage	Assessed as continuous variable	0.008	1.04 (1.01–1.06)	NS		0.006	1.04 (1.01–1.06)	NS	
Bone-marrow blasts >5%	231 (50.1)	0.003	1.46 (1.14–1.86)	NS		0.002	1.47 (1.15–1.89)	NS	
Circulating blasts ≥1%	77 (20.8)	0.009	1.51 (1.11–2.05)	0.003	1.63 (1.18–2.25)	0.031	1.41 (1.03–1.93)	0.011	1.51 (1.10–2.08)
eGFR <45 mL/min/1.73 m^2^	57 (13.0)	0.013	1.56 (1.10–2.21)	NS		0.009	1.60 (1.12–2.28)	NS	
Serum ferritin	Assessed as continuous variable	0.553	1.00 (0.99–1.01)	NS		0.352	1.00 (0.99–1.01)	NS	
Serum ferritin >200 μg/L	187 (53.6)	0.113	1.25 (0.95–1.66)	NS		0.300	1.16 (0.88–1.54)	NS	
Lactate dehydrogenase >246 U/L	159 (37.1)	0.027	1.34 (1.03–1.73)	NS		0.123	1.23 (0.95–1.59)	NS	
β2-microglobulin >3.0 mg/L	59 (40.7)	<0.001	2.62 (1.69–4.08)	NS		<0.001	2.52 (1.60–3.97)	NS	
ESA use	249 (94.0)	0.331	1.36 (0.73–2.50)	NS		0.516	1.24(0.65–2.35)	NS	
AZA use^d^	166 (35.5)	0.295	0.87 (0.68–1.12)	NS		0.193	0.85 (0.66–1.09)	NS	

*IPSS-R* revised international prognostic scoring system, *MDS-CI* myelodysplastic-syndrome specific comorbidity index, *WHO* World Health Organization, *IPSS* international prognostic scoring system, *FPSS* French prognostic scoring system, *EASIX* endothelial activation and stress index, *eGFR* estimated glomerular filtration rate, *ESA* erythropoiesis-stimulating agent, *AZA* azacitidine, 95% CI 95% confidence interval, *NS* not significant, *ND* not determined.

^a^According to the MDS-CI.

^b^Log2 EASIX is characterized by highly asymmetrical tails as indicated by histograms and quantile-quantile plots (Q-Q plots); inverse hyperbolic sine (arc-sine) transformations of EASIX scores indicate that linearity is disrupted with higher log2 EASIX values, suggesting that EASIX should be used in the multivariate analysis as a binary rather than as a linear variable.

^c^Red-cell transfusion dependence was defined as having at least one red-cell transfusion every 8 weeks over a period of 4 months, according to the WHO-based prognostic scoring system (WPSS).

^d^AZA was administered at a dose of 75 mg/m^2^ for either 7-consecutive days or 7 days with a weekend break (5-2-2 schedule) per cycle on 28-day cycles. Hematologic, bone-marrow and cytogenetic changes were assessed after six cycles of treatment. Treatment response was evaluated according to the 2006 International Working Group (IWG) response criteria in myelodysplasia.

Separate analysis for IPSS-R score values 3.5 (*n* = 153) and >3.5 (*n* = 315) revealed significant between-group differences in OS (Table 1). The overall actuarial probability of survival for patients with IPSS-R 3.5 was 71.4%, 46.9%, and 31.3% at one, two, and three years, respectively. In comparison, the corresponding survival rates for those with IPSS-R > 3.5 were 68.5%, 43.0%, and 25.2% (*p* = 0.039).

We were intrigued by the fact that the survival curves of patients with IPSS-R 3.5 and IPSS-R > 3.5 dispersed on the Kaplan-Meier plot (Fig. 1A) and wanted to test the hypothesis that patients with IPSS-R 3.5 might be classified as lower-risk. Taking advantage of our total registry (*n* = 2972), we developed probability estimates for predicting survival within various subgroups of patients. We constructed a classification tree model to select the category with the highest model-predicted probability for OS^7–9^. Tree-structured survival analysis confirmed that there was a significant difference in OS between patients with IPSS-R 3.0–3.5 and those with 4.0–4.5 (Supplementary Fig. S3a). Remarkably, a log2 EASIX value of 0.179, of all risk factors studied, was able to further distinguish patients with IPSS-R 3.5 who truly had lower-risk disease (i.e. similar to patients with IPSS-R 3.0) from those who showed similar outcomes to patients with IPSS-R 4.0–4.5 (*p* = 0.005) (Supplementary Fig. S3b).

**Fig. 1:**
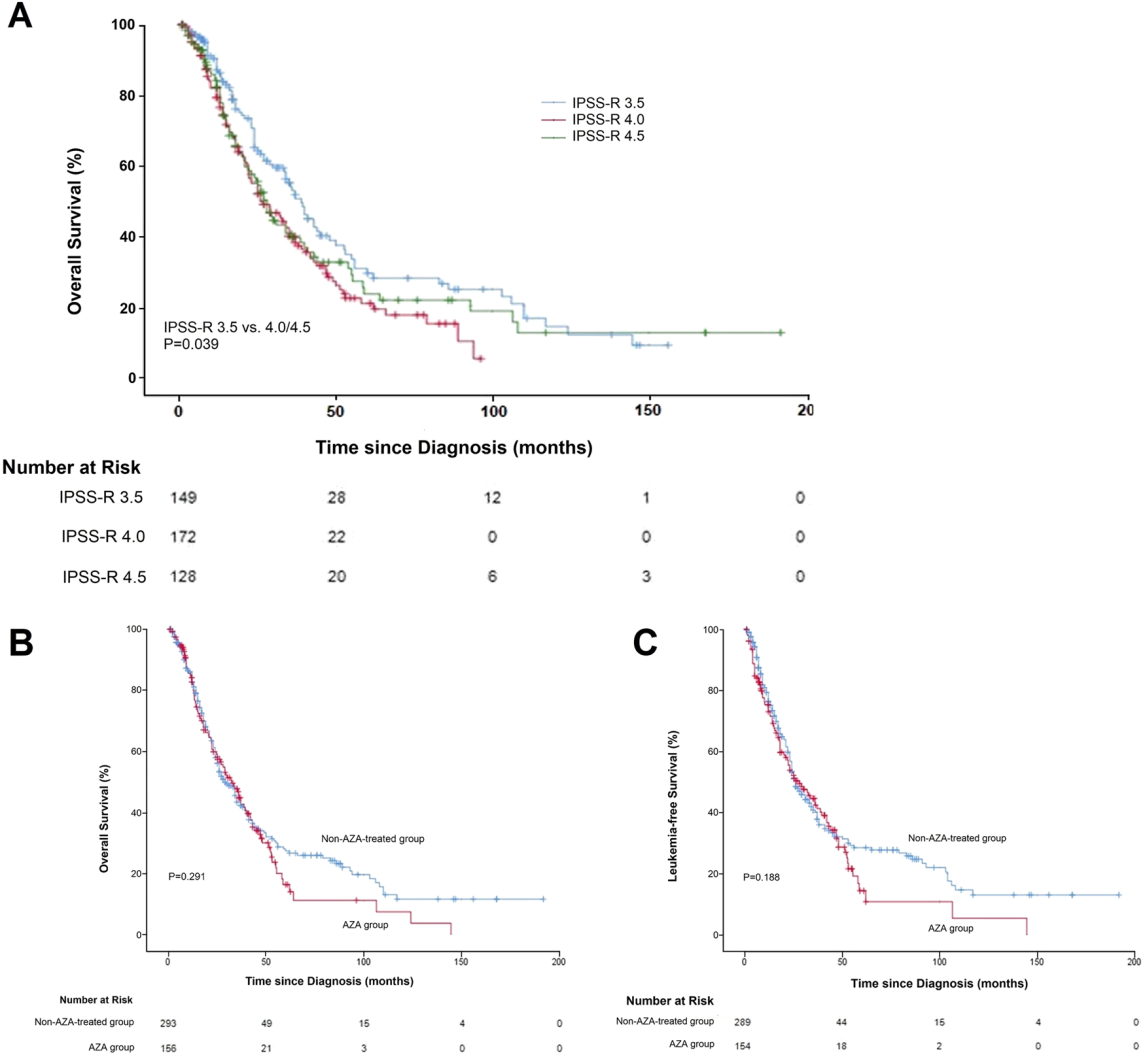
Kaplan–Meier analysis of survival outcomes in patients with intermediate-risk MDS. (**A**) Kaplan–Meier curves of OS for patients with intermediate-risk MDS according to their IPSS-R value (3.5, 4.0, or 4.5). OS was better among patients with IPSS-R 3.5 as compared with IPSS-R > 3.5 (*p* = 0.039 by the log-rank test). (**B**) Kaplan-Meier curve of OS among patients with intermediate-risk MDS who received AZA (*n* = 166), as compared with patients who did not (*n* = 302). Median OS of the patients who received AZA (32.4 months [95% CI 25.2–39.6]) was similar to patients who did not receive AZA (29.0 months [23.9–34.1]); *p* = 0.291 by the log-rank test. (**C**) Kaplan-Meier curve of LFS among patients with intermediate-risk MDS who received AZA (*n* = 166), as compared with patients who did not (*n* = 302). Median LFS was 28.0 months (95% CI 19.0–37.0) for the AZA group and 26.0 months (21.8–30.2) for the non-AZA-treated group (*p* = 0.188). MDS myelodysplastic syndrome, OS overall survival, IPSS-R revised international prognostic scoring system, AZA azacitidine, LFS leukemia-free survival.

Multivariate logistic regression was used to obtain odds ratios (OR) for AML transformation. Among the baseline factors evaluated, only the proportion of bone-marrow blasts (OR 1.16, 95% CI 1.09–1.23, for 1% increase in blast percentage; *p* < 0.001) and age (OR 0.98, 95% CI 0.96–1.00, for 1-year increase in age; *p* = 0.047) were independent predictors for eventual AML transformation. The overall risk of progression to AML was 52.8% at 4 years in patients with bone-marrow blasts >10% and 34.7% in those with ≤10% (*p* = 0.001). Supplementary Table S3 shows the characteristics of the patients who did not develop AML after >4 years.

We subsequently sought to investigate the role of AZA in intermediate-risk MDS. On average, patients in the AZA group (*n* = 166) were more likely to have severe anemia (*p* = 0.035), excess marrow blasts (*p* < 0.001), and higher-risk IPSS (*p* = 0.001), as compared with non-AZA-treated patients (Supplementary Table S4). Of the 166 AZA-treated patients, 16.3% achieved complete remission (CR) and 7.8% partial remission (PR). Αge, performance status, comorbidity, IPSS, cytogenetics, EASIX, eGFR, circulating and marrow blasts, and multilineage dysplasia had no appreciable influence on the chance of CR and/or PR.

Τhe median OS among patients receiving AZA (32.4 months [95% CI 25.2–39.6]) was similar to patients who did not receive AZA (29.0 months [23.9–34.1]), even after adjusting for hemoglobin, marrow blast count, IPSS, and IPSS-R (*p* = 0.291) (Fig. 1B). The results of subanalyses involving patients with low-risk disease (i.e. IPSS-R 3.5 with log2 EASIX < 0.179) and higher-risk disease (i.e. IPSS-R 4.0–4.5 and/or IPSS-R 3.5 with log2 EASIX > 0.179) showed no significant difference in OS according to the use or nonuse of AZA (*p* = 0.219 and *p* = 0.592, respectively) (Supplementary Fig. S4). Similarly, median LFS was 28.0 months (19.0–37.0) for the AZA group and 26.0 months (21.8–30.2) for the non-AZA-treated group (*p* = 0.188) (Fig. 1C). However, patients who achieved CR had significantly better survival than patients of matched age and sex who did not achieve CR (40.9 versus 29.4 months; *p* = 0.005) (Supplementary Fig. S5). Factors associated with worse outcomes in AZA-treated patients included response <CR (*p* < 0.001 for OS and LFS), age >70 years (*p* < 0.001 for OS; *p* = 0.007 for LFS), performance status ≥2 (*p* = 0.002 for OS; *p* = 0.004 for LFS), eGFR <45 mL/min/1.73 m^2^ (*p* = 0.002 for OS; *p* = 0.013 for LFS), and β2-microglobulin >3.0 mg/L (*p* < 0.001 for OS; *p* = 0.001 for LFS). After multivariate adjustment, only β2-microglobulin and response <CR remained significant. Beta-2-microglobulin presumably reflects subpopulations with renal impairment and/or excess blasts^10^. The results of a subgroup analysis involving patients at risk for shorter LFS (i.e. age >70 years and/or circulating blasts ≥1%) showed that the outcome was almost identical for those treated with AZA and for those not treated (*p* = 0.365 for OS; *p* = 0.399 for LFS).

Our study confirms that intermediate-risk IPSS-R may be considered as lower-risk if the score is 3.5 versus higher-risk if the score is >3.5. From a practical standpoint, this lends support to the NCCN MDS Practice Guidelines algorithm^11^. Most importantly, we showed that a single threshold value of log2 EASIX could be applied to further refine the IPSS-R 3.5 subgroup, and distinguish patients with low clinical risk from those with higher-risk disease. Though EASIX has been linked to endothelial dysfunction^6^, we did not find any association with major cardiovascular-disease events in our previous study (this study, however, did not include data on small-vessel damage owing to infectious and metabolic complications)^12^. Yet it may reflect other factors related to tumor biology, tumor burden, and host factors such as renal function. We regard EASIX as a valid tool complementary to the IPSS-R which should be prospectively evaluated as an additional classifier for patients with IPSS-R 3.5. The value of EASIX is particularly noteworthy in intermediate-risk patients with poor prognosis offered hematopoietic-cell transplantation, since it can be used to predict the patient’s individual risk of mortality after graft-versus-host disease and, potentially, indicate when therapies that reduce endothelial-cell damage are needed^6^.

Furthermore, we identified four simple, reproducible, and widely applicable risk factors as the strongest predictors of survival in intermediate-risk patients: age >70 years, peripheral blasts (≥1%), IPSS-R score >3.5, and log2 EASIX > 0.179. Further research is needed to determine the generalizability of these findings. In particular, the log2 EASIX cutoff point should be validated in independent external cohorts of patients. Another interesting point is that age and circulating blasts predicted for shorter LFS. Essentially, this suggests that the presence of circulating blasts is a marker of more aggressive biology.

Our findings and a previous report^13^ support the conclusion that AZA does not confer a survival benefit in intermediate-risk MDS. Our study also shows the major impact of CR on OS. This observation raises many interesting points. Patients should be informed of the small but real chance of CR (16.3%), as well as the small but real chance of a severe complication from AZA. To the clinician the all-important question would be how to identify patients who will go into CR. It must be emphasized that, in the present context, no biomarker exists for prediction of CR to AZA^14^.

Our study has some limitations. Data from observational studies may incorporate a degree of subjectivity and can be open to bias^15^. Owing to the retrospective study design, not all risk-factor variables were assessed in all patients. Therefore, the role of some variables in predicting outcomes might be underestimated.

The optimal treatment of intermediate-risk MDS remains an unmet medical need. If validated, the potential risk factors of older age, circulating blasts, IPSS-R > 3.5, and EASIX could aid early identification of patients with poor prognosis and indicate that a more intensive approach is needed, including hematopoietic-cell transplantation.

## Supplementary information

Supplementary Information

Author Checklist

## Originality statement

This manuscript contains original material that has not been published or submitted previously to another journal. All authors agree to the submission of this manuscript to the Blood Cancer Journal.
