# Supplementary material for: Refinement of prognosis and the effect of azacitidine in intermediate-risk myelodysplastic syndromes
Source: Blood Cancer J. 2021 Feb 11;11(2):30. doi: 10.1038/s41408-021-00424-4 (PMC7878783; doi:10.1038/s41408-021-00424-4)

**SUPPLEMENTARY INFORMATION**

**Supplement to:**

**Refinement of prognosis and the effect of azacitidine in intermediate-risk myelodysplastic syndromes**

Liapis K, Papadopoulos V, Vrachiolias G, Galanopoulos AG, Papoutselis M, Papageorgiou SG, Diamantopoulos PT, Pappa V, Viniou NA, Kourakli A, Τsokanas D, Vassilakopoulos TP, Hatzimichael E, Bouronikou E, Ximeri M, Pontikoglou C, Megalakaki A, Zikos P, Panayiotidis P, Dimou M, Karakatsanis S, Papaioannou M, Vardi A, Kontopidou F, Harchalakis N, Adamopoulos I, Symeonidis A, Kotsianidis I

**CONTENTS**

**Section 1: Statistical Analysis** ........................................................................................ Page 2

**Section 2: Supplementary Tables**

Supplementary Table S1 .............................................................................................. Page 4

Supplementary Table S2 .............................................................................................. Page 6

Supplementary Table S3 .............................................................................................. Page 7

Supplementary Table S4 .............................................................................................. Page 8

**Section 3: Supplementary Figures**

Supplementary Figure S1 .......................................................................................... Page 10

Supplementary Figure S2 .......................................................................................... Page 11

Supplementary Figure S3 .......................................................................................... Page 12

Supplementary Figure S4 .......................................................................................... Page 14

Supplementary Figure S5 .......................................................................................... Page 15

**SECTION 1: STATISTICAL ANALYSIS**

The primary outcomes were overall survival (OS) i.e. the time from the date of diagnosis of myelodysplastic syndrome (MDS) to the date of death due to any cause and leukemia-free survival (LFS) i.e. the time from MDS diagnosis to leukemic progression or death, whichever occurred first. We analyzed survival data, risk of progression to acute myeloid leukemia (AML) and median time-to-AML evolution for patients who received azacitidine and those who did not.

Univariate comparisons were performed with the use of Pearson’s *χ*^2^ or a two-sample independent t-test for discrete and normally-distributed continuous variables, respectively. To check the homogeneity of variance, we used Levene’s test. Kruskal-Wallis non-parametric test was preferred for small sample sizes (n≤20). Multivariate analysis of risk factors was performed with linear and binary logistic regression; every parameter that was significantly correlated in univariate analysis (p≤0.05) was treated as a potential independent parameter (the probability for stepwise entry and removal were set to 0.05 and 0.10, respectively; the classification cutoff was set to 0.5; and the maximum number of iterations was set to 20).

The association between patient and disease characteristics and an adverse outcome (disease progression or death) was assessed with the use of proportional-hazards models of time-to-death and time-to-progression to AML. Follow-up time was estimated by means of the reverse Kaplan-Meier method. We constructed Kaplan-Meier curves and used the log-rank test to determine the univariate significance of the study variables. We used a Cox proportional-hazards regression model to examine simultaneously the effects of multiple covariates on survival; OS and LFS were considered as dependent variables, whereas all other parameters were considered as potentially independent variables (the probability for stepwise entry and removal were set to 0.05 and 0.10, respectively; the classification cutoff was set to 0.5; and the maximum number of iterations was set to 20). In addition, we conducted a classification tree based analysis of time‐to‐event (survival) data. Classification tree analysis (CTA) is a decision-tree like classification model that provides accurate, sensitive and statistically robust decision rules that are easy to visually display and interpret. Classification-tree‒structured analysis uses optimal discriminant analysis (ODA) to identify the cutoff point on an ordered attribute (variable) by iterating through every value on the attribute and computing the effect strength for sensitivity (ESS); the optimal cutoff point is the one that yields the highest ESS versus all other cutoff points (see Reference 9 for a full description of classification trees and a comparison between the CTA framework and the Cox’s proportional-hazards model).

All reported p values were two-sided. The level of statistical significance was set to p=0.05; values of 0.05≤p≤0.1 were considered as requiring further evaluation. All numerical values are given with at least two significant digits. All analyses included data up to July 7, 2016. This nested design ensured that all patients were under observation throughout the entire study period, while also allowing us to assess the effects of several baseline risk factors on clinical outcomes.

All analyses were done with the use of IBM SPSS Statistics software, version 26.0, for Windows. MedCalc Statistical Software version 19.1 (MedCalc Software, Ostend, Belgium; 2019) was used for visualization of survival curves, and Review Manager version 5.3 (RevMan, Copenhagen: The Nordic Cochrane Centre, The Cochrane Collaboration; 2014) was used to illustrate forest plots.

**SECTION 2: SUPPLEMENTARY TABLES**

**Supplementary Table S1.** Clinical and hematologic characteristics of the 468 patients categorized as intermediate on the IPSS-R.

| **Characteristic** | **Value** |
| --- | --- |
| **Age** (years, median [range]) | 73 (40-92) |
| **Sex** — no. (%) |  |
| Male | 326 (69.7) |
| Female | 142 (30.3) |
| **Comorbidity*** — no. (%) |  |
| Cardiac | 93 (21.7) |
| Hepatic | 2 (0.5) |
| Pulmonary | 47 (11.0) |
| Renal | 17 (4.0) |
| **MDS-CI risk category** — no. (%) |  |
| Low | 255 (59.6) |
| Intermediate or high | 145 (33.9) |
| Unclassified | 28 (6.5) |
| **Performance status (WHO)** — no. (%) |  |
| 0 | 192 (49.5) |
| 1 | 141 (36.3) |
| 2 | 43 (11.1) |
| 3 | 12 (3.1) |
| ND | 80 |
| **WHO type** — no. (%) |  |
| Refractory cytopenia with unilineage dysplasia | 140 (32.6) |
| Refractory cytopenia with multilineage dysplasia | 154 (35.9) |
| Refractory anemia with excess blasts 1 | 85 (19.8) |
| Refractory anemia with excess blasts 2 | 34 (7.9) |
| Refractory anemia with ringed sideroblasts | 5 (1.2) |
| Unclassified | 4 (1.2) |
| Chronic myelomonocytic leukemia | 6 (1.4) |
| Inadequate specimen | 39 |
| **IPSS prognostic risk category** — no. (%) |  |
| Low | 17 (3.6) |
| Intermediate 1 | 372 (79.7) |
| Intermediate 2 | 77 (16.5) |
| High | 1 (0.2) |
| Unclassified | 1 |
| **IPSS-R score** — no. (%) |  |
| 3.5 | 153 (32.7) |
| 4.0 | 180 (38.5) |
| 4.5 | 135 (28.8) |
| **Cytogenetics^†^** — no. (%) |  |
| Very good | 9 (2.0) |
| Good | 325 (71.7) |
| Intermediate | 93 (20.5) |
| Poor | 17 (3.8) |
| Very poor | 9 (2.0) |
| **Erythrocyte transfusion dependence^‡^** — no. (%) |  |
| Yes | 178 (51.3) |
| No | 169 (48.7) |
| ND | 121 |
| **Hemoglobin** (g/dL, median [range]) | 9.5 (3.9-14.3) |
| **Absolute neutrophil count** (×10^9^/L, median [range]) | 1.6 (0.1-37) |
| **Platelet count** (×10^9^/L, median [range]) | 110 (10-1279) |
| **Bone-marrow blasts** (%, median [range]) | 5.4 (0-18) |
| **Circulating blasts** (%, median [range]) | 0 (0-12) |
| **eGFR** (mL/min/1.73 m^2^, median [range])**^§^** | 70 (14-137) |
| **Serum ferritin** (μg/L, median [range]) | 212 (10-32210) |
| **Lactate dehydrogenase** (U/L, median [range]) | 216 (75-1098) |
| **β2-microglobulin** (mg/L, median [range]) | 2.7 (0.8-17.9) |
| **Log2 EASIX** (median [range]) | 0.7 (-1.8-5.8) |

IPSS-R: revised international prognostic scoring system; MDS-CI: myelodysplastic-syndrome specific comorbidity index; WHO: World Health Organization; IPSS: international prognostic scoring system; eGFR: estimated glomerular filtration rate; EASIX: endothelial activation and stress index; ND: not determined.

*****According to the myelodysplastic-syndrome specific comorbidity index (MDS-CI); **†**According to the revised international prognostic scoring system; **‡**Red-cell transfusion dependence was defined as having at least one red-cell transfusion every 8 weeks over a period of 4 months, according to the WHO-based prognostic scoring system (WPSS); **§**Estimates of glomerular filtration rate (eGFR) were calculated using the Modification of Diet in Renal Disease or the Cockroft-Gault formulas, the latter used for patients weighing >25% beyond their ideal body weight.

**Supplementary Table S2.** Outcomes for the entire cohort of patients with intermediate-risk IPSS-R.

| **Follow-up period** (months) | **Value** |
| --- | --- |
| Median | 51.0 |
| 95% CI | 41.6-60.4 |
| **Overall survival** (months) |  |
| Median | 31.0 |
| 95% CI | 26.6-35.4 |
| **Leukemia-free survival** (months) |  |
| Median | 26.0 |
| 95% CI | 21.5-30.5 |
| **Time from MDS diagnosis to AML transformation** (months) |  |
| Median | 29.4 |
| 95% CI | 24.9-33.9 |
| **Rate of progression to AML** — no. (%) |  |
| At the end of the first year since diagnosis of MDS (n=390) | 83 (21.3) |
| At the end of the second year since diagnosis of MDS (n=389) | 116 (29.8) |
| At the end of the third year since diagnosis of MDS (n=389) | 133 (34.2) |
| At the end of the fourth year since diagnosis of MDS (n=389) | 144 (37.0) |
| At the end of the fifth year since diagnosis of MDS (n=389) | 147 (37.8) |

IPSS-R: revised international prognostic scoring system; MDS: myelodysplastic syndromes; AML: acute myeloid leukemia; 95% CI: 95% confidence interval.

**Supplementary Table S3.** Characteristics of the 245 intermediate-risk IPSS-R patients who did not have progression to AML after more than 4 years after diagnosis.

| **Characteristic** | **Value** |
| --- | --- |
| **Median age** | 74 years |
| **Sex** — no. (%) |  |
| Male | 165 (67.3) |
| **MDS-CI risk category** — no. (%) |  |
| Low | 143 (61.6) |
| **Performance status (WHO)** — no. (%) |  |
| 0-1 | 180 (87.8) |
| **WHO type*** — no. (%) |  |
| Refractory cytopenia with unilineage or multilineage dysplasia | 176 (79.6) |
| **IPSS prognostic risk category** — no. (%) |  |
| Lower risk | 213 (87.3) |
| **IPSS-R score** — no. (%) |  |
| 3.5 | 95 (38.8) |
| >3.5 | 150 (61.2) |
| **Cytogenetics^†^** — no. (%) |  |
| Very good or good | 264 (70.7) |
| **Erythrocyte transfusion dependence^‡^** — no. (%) |  |
| Yes | 112 (53.8) |
| **Median hemoglobin concentration** | 9.3 g/dL |
| **Median absolute neutrophil count** | 1.7×10⁹/L |
| **Median platelet count** | 100×10⁹/L |
| **Median bone-marrow blasts** | 4% |
| **Median circulating blasts** | 0% |
| **Median eGFR** | 69 mL/min/1.73 m^2^ |
| **Median lactate dehydrogenase** | 215 U/L |
| **Median β2-microglobulin** | 2.8 mg/L |

IPSS-R: revised international prognostic scoring system; AML: acute myeloid leukemia; MDS-CI: myelodysplastic-syndrome‒specific comorbidity index; WHO: World Health Organization; IPSS: international prognostic scoring system; eGFR: estimated glomerular filtration rate. *****According to the WHO 2008 classification; **†**According to the revised international prognostic scoring system; **‡**Red-cell transfusion dependence was defined as having at least one red-cell transfusion every 8 weeks over a period of 4 months, according to the WHO-based prognostic scoring system (WPSS).

**Supplementary Table S4.** Characteristics of patients with intermediate-risk MDS according to whether they underwent treatment with AZA. The ΑΖΑ group had a higher incidence of AML progression, possibly related to the higher proportion of patients with excess marrow blasts, higher-risk IPSS, and IPSS-R score >3.5.

| **Characteristic** | **AZA group**  (n=166) | **Non-AZA-treated group** (n=302) | **P value** |
| --- | --- | --- | --- |
| **Age** (years, median [range]) | 73.0 (48.0-92.0) | 73.0 (40.0-89.0) | NS |
| **Female sex** — no. (%) | 50 (30.1) | 210 (69.5) | NS |
| **Performance status (WHO) 2-4** — no. (%) | 14/130 (10.7) | 41/258 (15.9) | NS |
| **Cardiac comorbidity** — no. (%) | 34/144 (23.6) | 59/284 (20.8) | NS |
| **MDS-CI risk category** — no. (%) |  |  | NS |
| Low | 83/144 (57.6) | 172/284 (60.6) |  |
| Intermediate or high | 61/144 (42.3) | 112/284 (39.4) |  |
| Unclassified | 22 | 18 |  |
| **eGFR** (mL/min/1.73 m^2^) — no. (%) |  |  | NS |
| Median (range) | 71.8 (14.0-116.0) | 69.5 (17.0-137.0) |  |
| ≥45 | 17/154 (11.0) | 40/285 (14.0) |  |
| <45 | 137/154 (89.0) | 245/285 (86.0) |  |
| **WHO type** — no. (%) |  |  | <0.001 |
| RCUD/RCMD/RARS | 63/160 (39.4) | 236/273 (87.7) |  |
| RAEB 1/RAEB 2 | 86/160 (53.8) | 33/273 (12.1) |  |
| **IPSS prognostic risk category** — no. (%) |  |  | 0.001 |
| Low or intermediate 1 | 124/165 (75.1) | 265 (87.8) |  |
| Intermediate 2 or high | 41/165 (24.8) | 37 (12.3) |  |
| **IPSS-R score** — no. (%) |  |  | 0.009 |
| 3.5 | 41 (24.7) | 110/236 (36.4) |  |
| >3.5 | 125 (75.3) | 192/236 (63.6) |  |
| **Transfusion dependency** — no. (%) | 36/81 (44.4) | 142/266 (53.4) | NS |
| **Hemoglobin** (g/dL, median [range]) | 10.0 (4.3-14.0) | 9.3 (3.9-14.3) | 0.018 |
| **Absolute neutrophil count** (× 10^9^/L, median [range]) | 1.3 (0.1-37.0) | 1.8 (0.1-15.3) | NS |
| **Platelet count** (×10^9^/L, median [range]) | 113.0 (1.0-1279.0) | 103.0 (3.0-765.0) | NS |
| **Bone-marrow blasts** (%, median [range]) | 7.0 (0.0-18.0) | 5.0 (0.0-18.0) | <0.001 |
| **Number of peripheral blasts** (%, median [range]) | 0.0 (0.0-12.0) | 0.0 (0.0-5.0) | NS |
| **Serum ferritin** (μg/L, median [range]) | 212.0 (26.0-32210.0) | 2130 (9.0-3875.0) | NS |
| **Lactate dehydrogenase** (U/L, median [range]) | 215.5 (81.0-871.0) | 215.5 (75.0-1098.0) | NS |
| **β2-microglobulin** (mg/L, median [range]) | 2.7 (1.2-10.1) | 2.7 (0.8-17.9) | NS |
| **Log2 EASIX** (median [range]) | 0.7 (-1.7 – 5.8) | 0.7 (-1.8 – 5.0) | NS |
| **Overall survival** (months, median [range]) | 32.4 (25.2-39.6) | 29.0 (23.9-34.1) | NS |
| **Leukemia-free survival** (months, median [range]) | 26.0 (21.8-30.2) | 28.0 (19.0-37.0) | NS |
| **Progression to AML** — no. (%) | 60/116 (51.7) | 90/273 (33.0) | 0.001 |
| **Time from MDS to AML transformation** (months, median [range]) | 32.4 (25.3-39.6) | 28.0 (22.3-33.7) | NS |
| **FPSS score** — no. (%) |  |  | NS |
| Low | 24/68 (35.3) | 44/183 (24.0) |  |
| Intermediate or high | 44/68 (64.7) | 139/183 (76.0) | X |

MDS: myelodysplastic syndromes; AZA: azacitidine; AML: acute myeloid leukemia; IPSS: international prognostic scoring system; IPSS-R: revised international prognostic scoring system; MDS-CI: myelodysplastic-syndrome specific comorbidity index; WHO: World Health Organization; RCUD: refractory cytopenia with unilineage dysplasia (refractory anemia; refractory neutropenia; refractory thrombocytopenia); RAEB 1: refractory anemia with excess blasts 1; RAEB 2: refractory anemia with excess blasts 2; RARS: refractory anemia with ring sideroblasts; RCMD: refractory cytopenia with multilineage dysplasia; eGFR: estimated glomerular filtration rate; EASIX: endothelial activation and stress index; FPSS: French prognostic scoring system; 95% CI: 95% confidence interval; NS: not significant.

**SECTION 3: SUPPLEMENTARY FIGURES**

**Supplementary Figure S1. Hazard ratio and 95% CI for overall survival (OS) in patients with intermediate-risk IPSS-R.** Hazard ratios were estimated with univariate Cox regression models. IPSS-R: revised international prognostic scoring system; MDS-CI: myelodysplastic-syndrome‒specific comorbidity index; FPSS: French prognostic scoring system; EASIX: endothelial activation and stress index; eGFR: estimated glomerular filtration rate; 95% CI: 95% confidence interval.


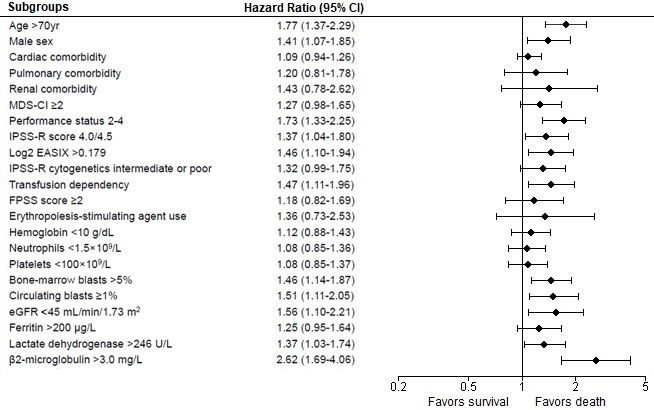


**Supplementary Figure S2. Hazard ratio and 95% CI for leukemia-free survival (LFS) in patients with intermediate risk IPSS-R.** Hazard ratios were estimated with univariate Cox regression models. IPSS-R: revised international prognostic scoring system; MDS-CI: myelodysplastic-syndrome‒specific comorbidity index; FPSS: French prognostic scoring system; EASIX: endothelial activation and stress index; eGFR: estimated glomerular filtration rate; 95% CI: 95% confidence interval.


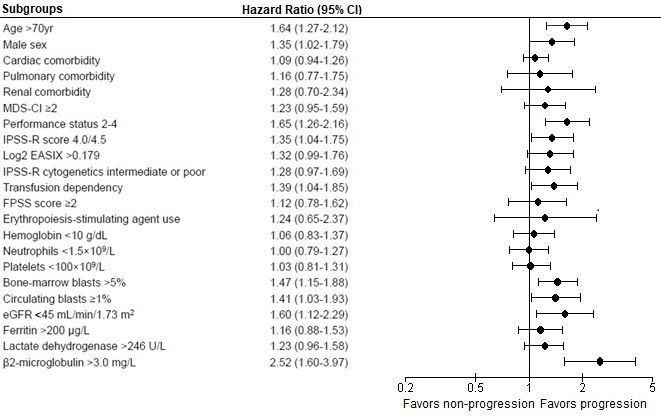


**Supplementary Figure S3.** **Classification tree model for the prediction of overall survival (OS) within various subgroups of patients with myelodysplastic syndromes.** **(a)** Taking advantage of our total registry (n=2972), we developed probability estimates for the prediction of survival within various subgroups of patients. We constructed a classification tree model to select the category with the highest model-predicted probability for OS, acknowledging that the modeled survival data converge with the Kaplan-Meier estimates of survival over long term follow-up. With this model, we calculated model-prediction statistics for five IPSS-R categories: category 1 (IPSS-R score ≤2.5); category 2 (IPSS-R 3.0-3.5); category 3 (4.0-4.5); category 4 (5.0-6.0); and category 5 (>6.0). This testing confirmed that there was a significant difference in OS between the patients with IPSS-R 3.5 and those with IPSS-R 4.0-4.5. On the basis of Kaplan-Meier estimates of OS, intermediate-risk patients with IPSS-R 3.5 were clustered in the same group along with patients with IPSS-R 3.0, whereas intermediate-risk patients with IPSS-R score values 4.0 or 4.5 showed significantly inferior OS. This means that patients classified as intermediate on the IPSS-R may be considered as lower risk if their score is 3.5 versus higher risk if their score is >3.5. **(b)** Decision tree classifier for the prediction of OS. Further subclassification of the patients with IPSS-R 3.5 was possible on the basis of the Endothelial Activation and Stress Index (EASIX). Optimal discriminant analysis was applied to test for a cutoff point in survival distributions with respect to EASIX levels; the optimal cutoff point was subsequently verified using standard maximally selected log-rank statistics. An EASIX cutoff point of 0.179 on the log2 scale could separate populations of significantly different OS (p=0.005). All other risk factors evaluated with respect to predicting OS such as the MDS-specific Comorbidity Index (MDS-CI), French Prognostic Scoring System (FPSS), International Prognostic Scoring System (IPSS), cytogenetic changes, marrow blast percentage, estimated glomerular filtration rate (eGFR), and serum β2-microglobulin lacked power to further subclassify patients in the IPSS-R 3.0-3.5 prognostic category. Thus the use of EASIX added meaningful prognostic information to the classification based on the IPSS-R. Interestingly, log2 EASIX had some prognostic value also in the IPSS-R 4.0-4.5 category but the level of statistical significance was borderline (p=0.048). EASIX is calculated as LDH (U/L) × creatinine (mg/dL) /platelet count (×10^9^/L). LDH: lactate dehydrogenase; IPSS-R: revised international prognostic scoring system; LN EASIX: log2 EASIX; Std. Dev.: standard deviation.


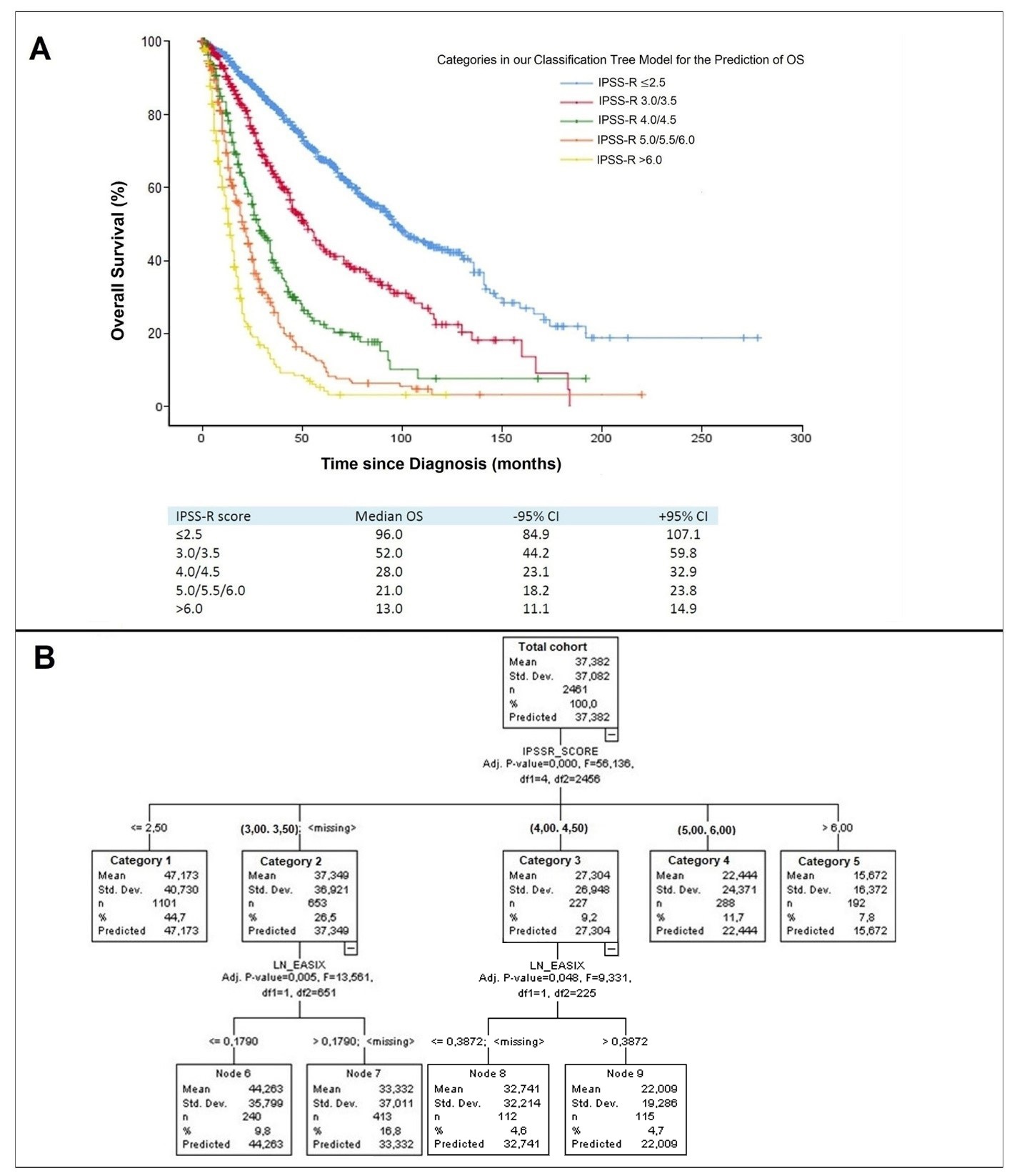


**Supplementary Figure S4.** **Kaplan-Meier curves of overall survival (OS) for intermediate IPSS-R patients with (a) lower clinical risk (i.e. IPSS-R 3.5 with log2 EASIX <0.179) and (b) higher clinical risk, with respect to whether they underwent treatment with azacitidine (AZA) or not**. There was no significant difference in OS according to the use or nonuse of AZA in any of the two subgroups (p=0.219 and p=0.592, respectively).

**
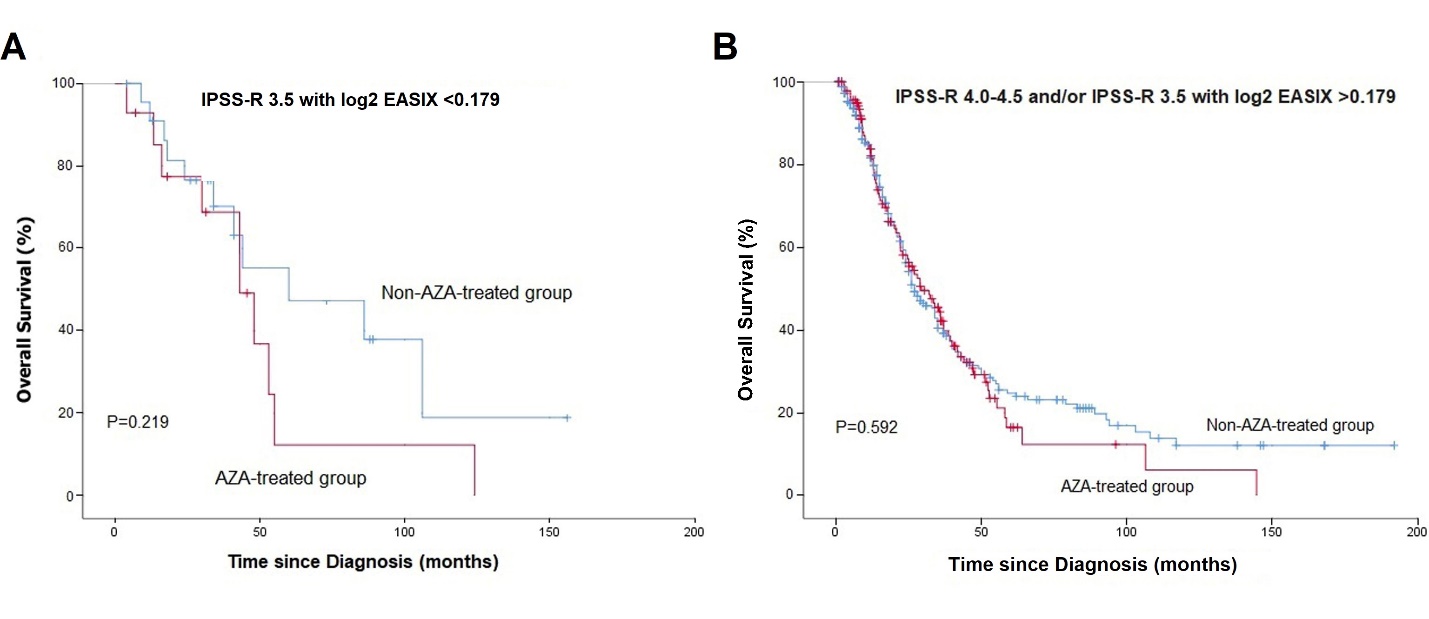
**

**Supplementary Figure S5.** **Kaplan-Meier curve of overall survival (a) and leukemia-free survival (b) of patients with intermediate-risk myelodysplastic syndromes who reached complete remission (CR) after treatment with azacitidine (AZA), as compared with patients who did not reach CR with AZA and patients who did not undergo treatment with AZA**. AZA conferred no obvious survival benefit beyond standard care, excluding patients in CR. The overall survival (OS) rate at 3 years was 75.0% among patients with CR and 27.3% among those with less than CR (p=0.001). Similarly, patients achieving CR had significantly better leukemia-free survival (LFS) than patients who did not achieve CR (55.3 versus 28.0 months; p=0.001). We have demonstrated that 16.3% of AZA-treated patients went into CR. We saw no evidence of different response rates to AZA between patients with IPSS-R 3.5 and >3.5. This means that the great majority of patients did not have a survival advantage with AZA. Mere improvement in the patient’s condition or hematologic status was not sufficient to significantly increase overall or leukemia-free survival.


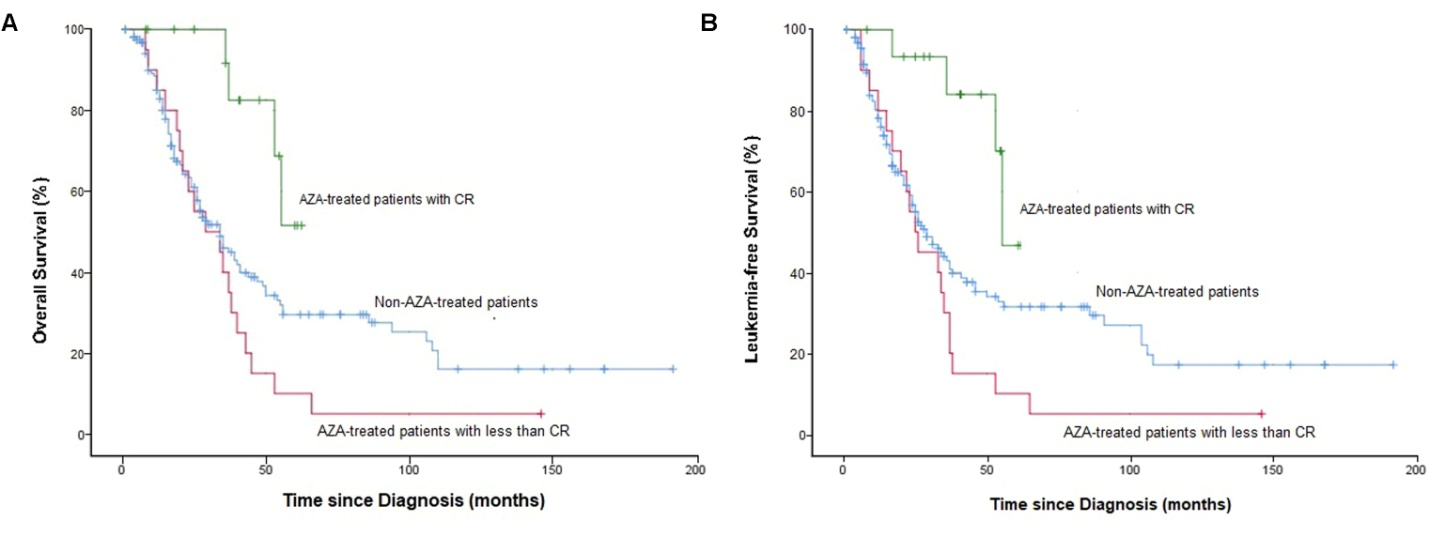

Supplement: Supplementary file 1 — Supplementary Information [file 41408_2021_424_MOESM1_ESM.docx]
